# Abatacept-Induced Panniculitis With Necrobiosis Lipoidica-Like Features in a Patient With Rheumatoid Arthritis

**DOI:** 10.7759/cureus.13460

**Published:** 2021-02-20

**Authors:** Scott Mahlberg, Thao Pham, Brittney DeClerck, Gloria J Stevens, Diem Q Pham

**Affiliations:** 1 Dermatology, Case Western University/University Hospitals Cleveland, Cleveland, USA; 2 Dermatology, Western University of Health Sciences, Lebanon, USA; 3 Dermatology/Dermatopathology, Keck School of Medicine at University of Southern California, Los Angeles, USA; 4 Dermatology, Keck School of Medicine at University of Southern California, Los Angeles, USA; 5 Dermatology, Western University of Health Sciences, College of Osteopathic Medicine of the Pacific, Pomona, USA

**Keywords:** panniculitis, abatacept, drug reaction

## Abstract

We present a 57-year-old female with a past medical history of rheumatoid arthritis, hypertension, and hypothyroidism who presented with poorly demarcated, nonblanching, painful, erythematous nodules on the bilateral lower legs for two weeks. The patient recently switched from infliximab to abatacept infusions, and skin eruptions presented 53 days from her initial abatacept infusion. A 5 mm punch biopsy of the left anterior upper leg in the zone of involvement showed a deep dermal granulomatous infiltrate with associated eosinophils and a vaguely horizontally palisaded pattern with necrobiosis. The granulomatous inflammation extended into the subcutaneous septae with a widening of the septae, edema, and lipomembranous fat necrosis. The patient was started on naproxen 500 mg PO BID and halobetasol propionate 0.05% lotion BID. Concomitantly, she was started on a four-day course of oral prednisone 10 mg PO daily and restarted infliximab infusions on the third day of prednisone treatment. At her initial infliximab infusions, she received one dose of solumedrol 40 mg and diphenhydramine 50 mg. The eruption resolved 21 days after the initial presentation. The present case is unique from the nine other cutaneous eruptions described after initiating abatacept therapy. Less than 10 cases of cutaneous panniculitides have been reported as adverse reactions to abatacept, with the most common reactions associated with oral contraceptives, nonsteroidal anti-inflammatory drugs, antibiotics, and leukotriene modifying agents. This case underscores the variety of histological findings in drug-induced panniculitis, highlighting the possibility of a drug reaction in a patient with rheumatological disease presenting with panniculitis.

## Introduction

Panniculitides are rare conditions that involve inflammation of the subcutaneous fat. The most prevalent subtype of panniculitis is erythema nodosum, with up to 55% of cases attributed to idiopathic etiologies [1]. Causes of panniculitis include infections, physical agents, autoimmune conditions, and malignancies [2]. Although the pathogenesis of panniculitis is not entirely understood, it is suspected that the underlying mechanism involves the deposition of immune complexes in the venules of septae of subcutaneous fat, triggering a neutrophilic inflammation. Histological findings will vary with different classifications and progression [1].

Abatacept (Orencia; Bristol-Myers Squibb, New York) emerged on the market on December 23, 2005, with indications for the treatment of moderate to severe rheumatoid arthritis in patients with inadequate response to disease-modifying antirheumatic drugs (DMARDs) or tumor necrosis factor-alpha (TNF-alpha) antagonists [3]. Upon literature review, 10 cases of cutaneous eruptions due to the usage of abatacept have been reported to date. Within these cases, one case of abatacept-induced panniculitis was reported with histological findings consistent with lupus erythematosus panniculitis (LEP) [4]. Nine other cutaneous eruptions described were not panniculitides and varied in histology [5].

Abatacept functions as a fusion protein, cytotoxic T-lymphocyte antigen (CTLA-4) linked to immunoglobulin G1 that blocks CD80 and CD86 ligands on antigen-presenting cells (APCs). This mechanism interferes with the CD28 binding site on T-cells, thereby down-regulating T-cell activation and decreasing inflammatory markers and cytokines [3]. In this case report, we present a paradoxical case of drug-induced panniculitis with necrobiosis lipoidica-like features, adding to the current literature surrounding cutaneous reactions associated with abatacept.

This article was previously presented as a meeting abstract at the American Academy of Dermatology conference on March 1, 2019.

## Case presentation

**Figure 1:**
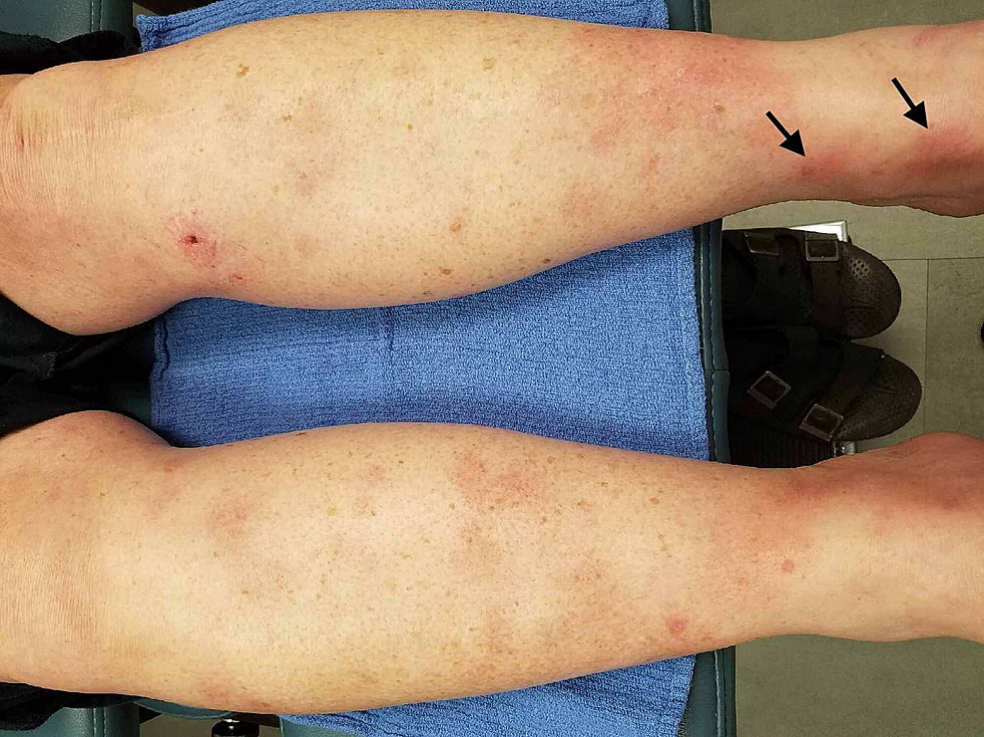
Poorly demarcated, nonblanching, painful, erythematous nodules on the bilateral lower legs.

A 57-year-old female with a past medical history of rheumatoid arthritis, hypertension, and hypothyroidism presented with poorly demarcated, nonblanching, painful, erythematous nodules on the bilateral lower legs for two weeks (Figure 1). Recently, the patient switched infusions from infliximab to abatacept. She received a total of three infusions of abatacept on days 0, 19, and 34; her rheumatologist discontinued treatment shortly after the presentation of her cutaneous lesions after her third infusion. She denied recent illness or trauma to the area.

**Figure 2:**
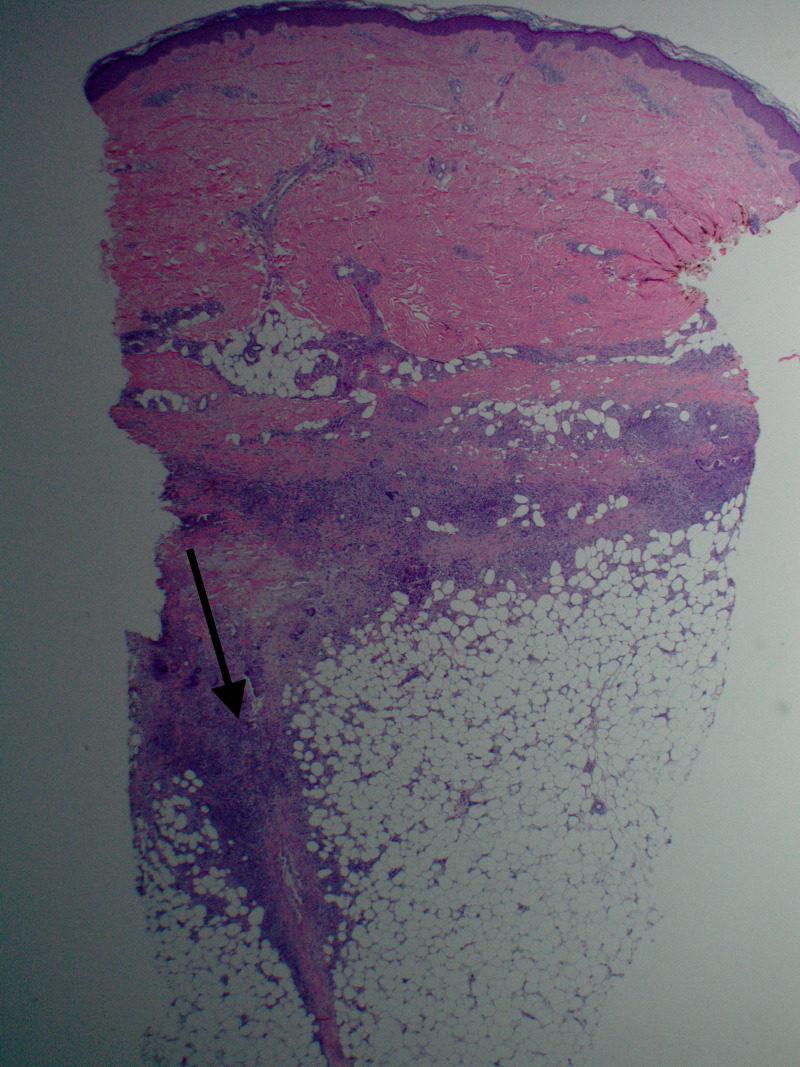
Granulomatous inflammation continued in the subcutaneous septae with a widening of the septae, edema, and lipomembranous fat necrosis.

The patient had been receiving infliximab for 20 years, she discontinued it five months before her presentation aiming for optimal management by trialing abatacept. Other medications included leflunomide for the previous two years, losartan for the past nine months, and a daily multivitamin. Punch biopsy of the left lower leg lesion showed a deep dermal granulomatous infiltrate with associated eosinophils and a vaguely horizontally palisaded pattern with necrobiosis. The granulomatous inflammation continued in the subcutaneous septae with a widening of the septae, edema, and lipomembranous fat necrosis (Figure 2). Acid-fast bacillus and fungal stains were negative.

Subsequently, the patient received naproxen 500 mg twice daily by mouth and halobetasol propionate 0.05% lotion topically twice daily. At her two weeks follow up for suture removal, her lesions were stable. She was subsequently started on a four-day course of 10 mg oral prednisone once daily. Infliximab was restarted on the third day of the prednisone course, with a one-time dose of 40 mg solumedrol and 50 mg IV diphenhydramine. Resolution occurred 21 days after the initial presentation.

## Discussion

A single drug may induce reactions with a variety of histological patterns, despite presenting with similar erythematous nodules [6]. The clinic-histological features of the present case require the consideration of erythema nodosum, rheumatoid nodules, necrobiosis lipoidica, subcutaneous granuloma annulare, and drug-drug interaction.

Cases of drug-induced panniculitis which appear throughout the literature are typically associated with oral contraceptives, NSAIDs, antibiotics, and leukotriene-modifying agents. The most common type of panniculitis caused by these agents is of the subtype erythema nodosum. Less commonly, agents such as glatiramer acetate, interferon beta, heparin, systemic steroids, tyrosine kinase, and BRAF inhibitors have been reported to induce panniculitis with histological findings consistent with lobular, mixed, or eosinophilic panniculitis with subcutaneous involvement [2]. Only one other case of panniculitis has been described after the initiation of abatacept have been reported, with features consistent with lupus panniculitis [4]. The histology of the present case was found to be compatible with panniculitis of mixed features, demonstrating deep dermal granulomatous inflammation with eosinophils and lipomembranous fat necrosis.

Classic histopathological findings of erythema nodosum include septal lymphohistiocytic inflammation without vasculitis [1]. Our patient’s biopsy showed palisaded granulomatous inflammation in the subcutaneous septae and lipomembranous fat necrosis, with deep dermal granulomatous inflammation with eosinophils, features consistent with panniculitis with necrobiosis lipoidica-like features. Although these findings are similar to necrobiosis lipoidica, the absence of a history of diabetes and the abrupt onset of symptoms in our patients makes this less likely to be a strict de novo necrobiosis lipoidica, which usually gradually enlarges over months to years.

Rheumatoid arthritis may rarely present with cutaneous manifestations in up to 15% of patients. Twenty percent of skin lesions present late in the course of rheumatoid arthritis as rheumatoid nodules [7]. Rheumatoid nodules are more likely to occur on joint surfaces and are well-demarcated compared to the nodules described in this case. Rheumatoid nodules have been reported to develop in patients during therapy with certain drugs such as methotrexate and biologics. On histology, central necrosis within the dermis surrounded by palisaded macrophages, and a perivascular infiltrate of inflammatory cells in the outer regions in the dermis are commonly seen [8]. Our patient had discontinued infliximab five months prior to the presentation of her lesions on her lower extremities and was not treated with methotrexate. Histological finding of her biopsy differs from those of a typical rheumatoid nodule, with associated eosinophils and the absence of significant mucin deposition. Her biopsy also did not show perivascular infiltrate of inflammatory cells typically seen in rheumatoid nodules, making this diagnosis less likely.

Although similar to histological findings to the present case, subcutaneous granuloma annulare is usually nontender and would more likely present in a child. Drug interaction between leflunomide and abatacept was considered; however, no prior cases of panniculitis precisely due to the interaction of the medications, as mentioned above, have been reported. In the Abatacept Study of Safety in Use with Other Rheumatoid Arthritis therapies (ASSURE) trial, 0.9% of patients who concomitantly took abatacept and leflunomide experienced a skin reaction. There is neither clinical or histopathological description of these adverse effects in the report, nor is there a distinction between injection site reactions or generalized skin reactions that may occur in infusion-related reactions [9-10]. Therefore, a drug-drug interaction cannot be entirely eliminated.

Based on the mechanism of abatacept and the inflammatory pattern seen on histology, it is peculiar that a T-cell inhibitor would cause a histiocytic response. Many drug reactions are poorly understood, including the paradoxical drug reactions described with TNF-inhibitors like adalimumab [11]. The present case’s histological features support a Type IVa reaction pattern, driven by histiocyte secretion of IFN-gamma and the induction of granulomatous inflammation [12]. One hypothesis may involve the patient’s discontinuation of infliximab, a TNF-alpha inhibitor, and a drug reaction similar to immune reconstitution inflammatory syndrome [13]. Removing the suppression of TNF-alpha, followed by a drug reaction, could cause excessive stimulation of histiocytes through a rebounded TNF-alpha inflammatory response, resulting in the secretion of IL-12. This could then drive a Th1 response promoting the IFN-gamma secretion resulting in the Type IVa drug reaction described above.

Accounting for these differential diagnoses, the time-frame of presentation, and the resolution upon discontinuing the medication, the Naranjo adverse drug reaction probability scale was utilized to calculate a total score of 6, indicating a probable reaction to abatacept (2-Appeared after drug administered + 1-Improved upon discontinuation + 2-Unlikely other causes resulted in this reaction + 1-Confirmed on objective biopsy) [14].

## Conclusions

In this report, we have presented a case of abatacept-induced panniculitis with necrobiosis lipoidica-like features, which is clinically similar, though histologically distinct from previously described abatacept reactions. Erythema nodosum, rheumatoid nodules, necrobiosis lipoidica, subcutaneous granuloma annulare, and drug-drug interaction were considered in this case. However, the patient’s age, clinical presentation, and histological features are most consistent with the diagnosis of panniculitis with necrobiosis lipoidica-like features.

Using the Naranjo adverse drug reaction probability scale, we calculated a total score of 6, indicating a probable reaction to abatacept. This case of abatacept induced panniculitis with necrobiosis lipoidica-like features should bring awareness to providers of the potential cutaneous reaction relating to abatacept. It will also be useful in hypothesizing the mechanism of biologic-induced reactions of all patterns and add to clinicians’ differential for cutaneous panniculitides as the use of biologic agents continues to rise.
